# Bypassing misinformation without confrontation improves policy support as much as correcting it

**DOI:** 10.1038/s41598-023-33299-5

**Published:** 2023-04-12

**Authors:** Christopher Calabrese, Dolores Albarracín

**Affiliations:** 1 Department of Communication, Clemson University, Clemson, SC 29634 USA; 2 The Annenberg Public Policy Center, University of Pennsylvania, Philadelphia, PA 19104 USA

**Keywords:** Psychology, Human behaviour

## Abstract

Curbing the negative impact of misinformation is typically assumed to require correcting misconceptions. Conceivably, however, bypassing the misinformation through alternate beliefs of opposite implications may reduce the attitudinal impact of the misinformation. Three experiments, one preregistered with a sample representative of the United States population, examined the impact of (a) directly correcting prior misinformation offered in support of restricting Genetically Modified (GM) foods (i.e., the correction strategy) and (b) discussing information in support of GM foods (i.e., the bypassing strategy), compared to a misinformation-only control condition. Findings consistently revealed that bolstering beliefs with opposite implications is just as effective at reducing opposition to GM foods as is correcting misinformation about GM foods. Thus, bypassing should be added to our arsenal of methods to curb the impact of misinformation.

## Introduction

Even though debunking, which focuses on correcting a belief after exposure to the misinformation^1^, is generally successful at mitigating misinformation, human beings do not like to be corrected and some corrections may increase memory for the misinformation^2^. Furthermore, certain beliefs, including conspiracy theories, are not falsifiable and can be difficult to debunk^3^. We then ask: What are other ways of addressing the impact of misinformation? How could we reduce the negative impact of misinformation about a new technology on support for policies concerning that technology?

Answering this question requires a psychological understanding of how beliefs and attitudes are organized. A belief is a subjective estimate that Genetically Modified (GM) foods, for example, are likely to bring about negative outcomes, such as cancer. An attitude is the evaluation of the object, in this case GM foods, as positive or negative, and may impact support for a policy restricting GM foods. In thinking about the relation between attitudes and beliefs, two distinct possibilities arise. On the one hand, attitudes can be linked to a single belief such as the estimated likelihood that GM foods cause cancer, a clear negative outcome (for models of such a situation, see^4,5^. Given this structure, correcting such a negative belief should be essential to reduce anti-GM food policies. On the other, attitudes can be linked to multiple beliefs^6^. Given the limits of human cognitive capacity, these multiple beliefs will rarely be activated at the same time^4,7^. In this case, intervening to highlight a belief that reduces support for anti-GM food policies should be the way to go.

We propose *bypassing* as a new perspective for addressing misinformation. The bypassing strategy involves redirecting an individual’s attention away from the misinformation to other beliefs, either bolstered or introduced a new, that support a conclusion opposite to the conclusion that can be drawn from the misinformation. For example, a policy maker trying to curb misinformation that GM foods cause cancer may introduce information that negates the GM-food-cancer link. Alternatively, however, the policy maker may instead bolster a common belief in the benefits of GM foods for the environment or introduce a new belief in the benefits of GM food for saving the bees. This approach capitalizes on understanding attitudes as based on different beliefs, avoids focusing on the misinformation as the sole basis for attitudes in a controversial area, and centers around the conclusion or outcome of the misinformation itself.

In three studies, we experimentally tested the effects of bypassing and correcting misinformation compared to a misinformation-only condition. The data and codes from our studies are available in https://osf.io/rgw84/?view_only=18c30e212c97419e95e917e326e810a5. Participants read news articles about GM crops. In Experiments 1 and 2, they received misinformation that described GM corn products as causing severe allergic reactions. After reading the misinformation, participants were randomly assigned to (a) a second article that highlighted a positive belief about GM crops, (b) a second article that corrected the misinformation using factual refutation along with an alternative explanation, or (c) a control condition in which participants did not receive a second article (Experiment 1) or read an unrelated control article on airport travel (Experiment 2). In Experiment 3, participants were randomly assigned into one of four conditions: three of the four conditions involved a misinformation article that discussed a well-known, albeit retracted study in which GM corn was linked to tumor growth in mice, whereas the fourth condition contained two unrelated articles on airport travel and parks to serve as a true, no-misinformation control.

In Experiment 1, the bypassing condition involved the belief that GM crops may help alleviate global hunger and malnutrition^8^. In Experiments 2 and 3, the bypassing condition focused on the beliefs that GM crops can help save the bee population^9^. It is important to note that the implementation of GM crops to achieve these benefits is nuanced. For example, the development of GM crops to resist drought and disease or reduce the need for tillage can potentially alleviate global hunger and improve insect pollinator populations. However, biotech company ownership of GM seeds and their potential control over food supply, as well as the higher costs of GM seeds, may hinder the use of GM crops to alleviate global hunger and improve insect populations. Despite these concerns, evidence does suggest that GM foods can be developed to be drought, disease, and pest-resistant, which can ultimately increase food production for humans and nesting habitats for insect pollinators.

We measured both beliefs in the misinformation and beliefs in information supporting conclusions opposite of the misinformation. (Belief in accurate information, opposite to the misinformation, which were measured as well, can be found in Supplemental Materials due to space constraints). In Experiments 1 and 2, we measured the belief in misinformation that a newly developed GM corn product causes severe allergic reactions (1 = not at all to 5 = very much so). In Experiment 3, we measured the belief in misinformation that GM corn accelerates tumor growth in rats (1 = not at all to 5 = very much so). With respect to the beliefs with implications opposite to the misinformation, in Experiment 1, beliefs in the conclusion opposite of the misinformation were measured with 2-items that assessed participants’ agreement with the belief that the production of *GM foods can alleviate global hunger/is key to feeding our global population* (1 = not at all to 5 = very much so). These beliefs are quite salient in the US population but may be used in judgment only when participants are reminded of it. Thus, they may be similarly strong across conditions but drive policy support and attitudes only in the bypassing condition. In Experiments 2 and 3, the bypassing beliefs were measured with 2-items that assessed participants’ agreement with the beliefs, *GM crops are beneficial for the bee population/are key to saving the bee population* (1 = not at all to 5 = very much so). These beliefs are novel and therefore should differ across bypassing and other conditions.

## Results

### Experiment 1

We were interested in examining if simply advocating for GM foods could successfully remove the effect of the initial misinformation, just as a correction might do. Thus, for Experiment 1 we recruited 360 participants from Prolific (www.prolific.co). Experiment 1 included three conditions. The first condition was misinformation followed by information on a belief of implications opposite to the misinformation, which served the purpose of bypassing the earlier concerns the misinformation had raised. The second was misinformation followed by a correction in which the misinformation claim was denied and evidence in support of the denial was provided. The third was a misinformation-only group, which served as a control to be compared with the two methods of curbing the impact of the misinformation on support for GM food restrictions. In all conditions, participants were debriefed about the study and read a statement providing accurate information directly refuting the misinformation they read.

The results from this experiment appear in Table 1. As shown, both bypassing and correction led to weaker intentions to support GM food restrictions and less positive attitudes toward those restrictions than did the control condition. Thus, we demonstrated that in addition to correcting misinformation by directly confronting, the misinformation may be simply bypassed with a similarly favorable outcome.

**Table 1:** Experiment 1 results (N = 360).

	Misinformation & bypassing *M* (SD)	Misinformation & correction *M* (*SD*)	Misinformation only (control) *M* (SD)	*F* (2, 357)	P
Intention to support policy restrictions on the production of GM foods	2.19^a^ (1.29)	2.26^a^ (1.19)	2.63^b^ (1.32)	4.29	0.014
Attitude toward supporting policy restrictions on GM foods	3.37^a^ (1.82)	3.63^a^ (1.79)	4.30^b^ (2.08)	7.67	0.001
Belief in the misinformation about GM foods	2.18^a^ (1.13)	1.61^b^ (0.88)	2.27^a^ (1.18)	13.31	< 0.001
Bypassing beliefs that GM foods alleviate global hunger	3.39^a^ (1.25)	3.42^a^ (1.17)	3.22^a^ (1.31)	0.933	0.394

Different superscripts indicate statistically significant pairwise contrasts.

Experiment 1 also measured the beliefs in the misinformation as well as the beliefs bolstered in the bypassing condition. We found large effects of the correction on the belief in the misinformation and no effects on the bolstered beliefs. The lack of significant effects on the bolstered beliefs suggests that the beliefs introduced in the bypassing condition were present in all conditions even though reminding participants of them was clearly impactful.

All in all, Experiment 1 indicated that redirecting people’s attention to beliefs whose implications counter the misinformation reduces the negative impact of the misinformation. Our findings also suggest that the belief used in the bypassing condition—that GM foods to help feed our growing population and alleviate malnutrition—was already present in the minds of our participants. Therefore, we were interested in finding out if introducing a *new* bypassing belief could also decrease support for GM food restrictions. Therefore, we ran a second experiment with a new bypassing belief that GM foods can help save bees in our planet. In addition, the misinformation control condition in the second experiment included the earlier misinformation article followed by another article about airport travel. Thus, this control condition could test the robustness of our effects on policy support and attitudes after more precisely equating the length of the information presented across conditions.

### Experiment 2

In Experiment 2, we recruited 303 participants from Prolific (www.prolific.co) and utilized a new bypassing condition that highlighted the potential for GM foods to help save the bee population. The results, which appear in Table 2, again showed that both the bypassing and the correction conditions significantly reduced support for GM food restrictions and attitudes toward supporting these policies than did the control condition.

**Table 2:** Experiment 2 results (N = 303).

	Misinformation & bypassing *M* (*SD*)	Misinformation & correction *M* (*SD*)	Misinformation control *M* (*SD*)	*F* (2, 300)	p
Intention to support policy restrictions on the production of GM foods	2.45^a^ (1.30)	2.50^a^ (1.35)	2.98^b^ (1.41)	4.60	0.011
Attitude toward supporting policy restrictions on GM foods	3.76^a^ (1.90)	3.91^a^ (2.11)	4.57^b^ (2.02)	4.64	0.010
Belief in the misinformation about GM foods	2.47^a^ (1.26)	1.84^b^ (1.24)	2.96^c^ (1.33)	19.74	< 0.001
Bypassing belief that GM foods will save the bee population	3.42^a^ (1.18)	2.16^b^ (1.06)	2.01^b^ (0.93)	53.38	< 0.001

Different superscripts indicate statistically significant pairwise contrasts.

As in Experiment 1, the findings from this study, which appear in Table 2, revealed that the correction was highly effective at weakening the belief in the misinformation consistent with the misinformation. Specifically, relative to the misinformation control condition and the bypassing condition, the correction condition led to weaker beliefs that GM foods cause allergies. In addition, the bypassing condition led to stronger beliefs in the ability of GM foods to save the bee population, which was a new belief introduced in that message. As shown, relative to the misinformation control and the correction conditions, the bypassing one led to stronger beliefs that GM foods have a positive impact on the bee population.

In conclusion, Experiments 1 and 2 provided consistent results about the effectiveness of bypassing in reducing the policy support and attitudinal effects of misinformation. However, it was important to further verify that the misinformation was effective and neither of the first two experiments included a control condition without the misinformation. Thus, Experiment 3 added a no-information control to serve as an additional baseline. Further, we also utilized a new misinformation claim about GM crops to increase the external validity of our findings, preregistered the study, and reached a sample representative of the United States population.

### Experiment 3

Our last experiment focused on the misinformation claim that GM crops accelerated tumor growth. In this preregistered study (https://aspredicted.org/blind.php?x=BJJ_3DSwe) we recruited 772 participants from Dynata (www.dynata.com). The sample, as shown in Supplementary Table 1, approximated the Census in terms of gender, race/ethnicity, and education. The results from this experiment, which appear in Table 3, showed that the misinformation had indeed increased support for GM food restrictions relative to the no-misinformation control.

**Table 3:** Experiment 3 results (preregistered, N = 772).

	Misinformation & bypassing *M* (*SD*) or %	Misinformation & correction *M* (*SD*) or %	Misinformation control *M* (*SD*) or %	No misinformation control *M* (*SD*) or %	*F*	p
Intention to support policy restrictions on the production of GM foods	3.27^a^ (1.20)	3.29^a^ (1.22)	3.58^b^ (1.14)	3.08^a^ (1.21)	5.72	.001
Attitude toward supporting policy restrictions on GM foods	4.89^a^ (1.75)	4.89^a^ (1.84)	5.34^b^ (1.55)	4.80^a^ (1.67)	3.62	.013
Belief in the misinformation about GM foods	81%^a^	56%^b^	76%^a^	59%^b^	–	–
Bypassing belief that GM foods will save the bee population	72%^a^	40%^b^	38%^b^	48%^b^	–	–

Different superscripts indicate statistically significant pairwise contrasts.

Similar to the previous experiments, both the bypassing and the correction conditions led to weaker support for GM food restrictions than did the misinformation condition. The bypassing and correction strategies also led to less positive attitudes toward GM restrictions than did the misinformation condition (Table 3). Furthermore, policy support and attitudes in the bypassing and correction conditions did not differ from the no-misinformation control, indicating that both conditions successfully eliminated the impact of the misinformation.

As in the other experiments, we also measured the belief in the misinformation along with the bypassing belief that GM foods would save the bee population. This time, however, those beliefs were measured dichotomously to better visualize the impact of the different strategies. Consistent with Experiment 2, we find that the correction condition reduced belief in the misinformation compared to the misinformation control condition (OR = 0.40, 95% CI [0.25, 0.62]). In addition, we find that the no-misinformation control had a significantly lower belief in misinformation compared to the misinformation control (OR = 0.45, 95% CI [0.28, 0.69]), indicating the importance of addressing the impact of the misinformation. We also find that the bypassing condition produced stronger beliefs that the production of GM crops can help save the bee population compared to the misinformation control (OR = 4.17, 95% CI [2.72, 6.46]), consistent with Experiment 2.

## Discussion

At a time when misinformation represents a threat to health, political, and social outcomes, finding ways of addressing its impact on citizens’ attitudes and policy support is critical. The research we conducted here is, to the best of our knowledge, the first to demonstrate the efficacy of addressing misinformation without direct confrontation, by shifting the focus from the misinformation to the outcome of the misinformation. This approach capitalizes on the capacity of a human mind to form networks of beliefs in which more than one belief can affect attitudes and ultimate policy support. Rather than focusing on revising beliefs, we found that this bypassing strategy is effective at highlighting an alternate belief and affecting the attitudes of an audience without invalidating the misinformation.

Both the bypassing and correction strategies impacted attitudes and policy support, and future work should examine in what contexts the bypassing approach is more successful at curbing misinformation than corrections. For example, the bypassing strategy may be more effective than corrections when the corrections threaten important aspects of the recipients’ identity. Also, individuals who identify with biotech conspiracy groups or hold strong negative attitudes toward GM foods may resist being confronted about their beliefs in a biotech conspiracy. In these cases, emphasizing new supportive information about GM foods may be more effective than attempting to revise conspiracy beliefs. A caveat is that when the misinformation is high in accessibility, bypassing may not manage to redirect attention, in which cases other approaches may be necessary.

This research is not without limitations. Our studies examined strategies to combat misinformation without examining long term impacts, though our studies included a short delay between exposure to the stimuli and outcome measurement. Further research should examine bypassing as a strategy to address other health, science, and political-related misinformation, in both lab and field experimental settings, in the context of multiple messages, and over a longer period of time. Further, even though our study demonstrated the impact of the strategy on both policy support and attitudes, future research should examine the impact of bypassing misinformation on behaviors. However, the innovation this work offers lies in changing our perspective of how to impact attitudes and policy support without direct correction. Given people’s limits in their ability to revise their beliefs, this alternate path of least resistance could be quite consequential.

## Materials and methods

### Experiment 1

#### Design and participants

All experimental protocols were reviewed and approved by the Institutional Review Board of the University of Pennsylvania and all participants provided informed consent. In addition, all research was carried out in accordance with relevant guidelines and regulations. We recruited 360 participants from Prolific (www.prolific.co) for a between-subjects experiment with three conditions. Each participant was asked to read one or two articles of relatively equal word length (208–215 words). Each article included a title, image, and main text to mimic a real news article. First, all participants read an article containing misinformation about GM foods. The article describes a false narrative that the ingestion of GM corn products led to severe allergic reactions and expressed caution about the dangers of GM foods. Then, participants were randomized to one of three conditions: (1) bypassing condition, (2) correction condition, and (3) misinformation control. Participants in the bypassing condition read an article describing the potential of developing GM foods to alleviate global hunger. The article described several ways in which GM crops may be developed to feed the world population. Those in the correction condition read an article that directly addressed the misinformation in the previous article. The article utilized best practices by providing an alternative explanation and factual refutation of the misinformation. Those in the misinformation control condition did not read a second article. All participants then answered questions assessing our outcome variables. After completing the questionnaire, all participants were debriefed about the study and read a statement providing correct information about GM foods and directly refuted the misinformation they read in the first article.

On average, participants were 34.56 (*SD* = 12.27) years old and about 48% identified as female, 50% identified as male, and 2% identified as another gender. Most participants identified as White (83%), while 8% identified as Asian, 3% identified as Black or African American, 1% identified as American Indian or Alaskan Native, 4% identified as multiracial, and 1% identified as other race. About 11% identified as Hispanic or Latino. About 56% of participants had a bachelor’s degree or higher, 54% were single, and the median income was $50,000 to $59,999. Further, the sample was relatively liberal (M = 2.98, SD = 1.58) and 53% identified as a Democrat. Although most participants indicated no religion or religious beliefs (54%), about 18% identified as Protestant and 14% identified as Roman Catholic.

#### Measures

Our primary dependent variable was intention to support policies that restrict the production of GM foods. We measured policy support intentions (from 1 = not at all likely to 5 = extremely likely) with two items that asked, *How likely would you support policies that restrict the manufacturing/production of genetically modified (GM) foods?* The items were averaged and were highly correlated (*r* = 0.92).

The second dependent variable was attitudes toward the policies. We measured attitudes using a 7-point semantic differential scale with the question, *In general, policies that restrict the manufacturing of genetically modified (GM) foods are…* with *Bad/Good* and *Unhelpful/Helpful* as responses. The two items were averaged and were highly correlated (*r* = 0.94).

We also examined belief in the misinformation using a 1-item measure with the question, *I believe that the newly developed genetically modified (GM) corn causes severe allergic reactions* from 1 = not at all to 5 = very much so.

Lastly, we assessed the bypassing belief, which was measured by averaging two items from not at all to very much so. These items include, I believe that genetically modified (GM) foods can help alleviate global hunger and malnutrition/The production of genetically modified (GM) foods is key to feeding our global population. The reliability of the measure was satisfactory (α = 0.88).

#### Analysis

We utilized ANOVA with planned contrasts to assess experimental effects on all outcome variables.

### Experiment 2

#### Design and participants

We recruited 303 participants from Prolific (www.prolific.co) for a between-subjects experiment with three conditions. Experiment 2 was identical to Experiment 1 except for the contents in the bypassing and misinformation control conditions. In this experiment, participants in the bypassing condition read an article describing the potential for GM crops to save the bee population. The article described several ways in which GM crops can be developed to help insect pollinators prosper. In addition, those in the misinformation control condition read a control article on airport traveling. After reading the two articles, participants completed our dependent measures. Lastly, all participants were debriefed about the study and read a statement providing correct information about GM foods and directly refuting the misinformation they read in the first article.

On average, participants were 36.50 (SD = 14.37) years old and about 50% identified as female, 49% as male, and 1.0% as another gender. Most participants identified as White (69%), while 11% identified as Asian, 7% identified as Black or African American, less than 1% identified as American Indian or Alaskan Native, 8% identified as multiracial, and 5.0% identified as other race. About 10% identified as Hispanic or Latino. About 53% of participants had a bachelor’s degree or higher, 56% were single, and the median income was $50,000 to $59,999. Further, the sample was relatively liberal (M = 2.95, SD = 1.72) and 50.1% identified as a Democrat. Although most participants indicated no religion or religious beliefs (54%), about 13% identified as Protestant and 16% identified as Roman Catholic.

#### Measures

The measurement procedures in this experiment were the same as in Experiment 1 except for the bypassing beliefs. Using measures identical to Experiment 1, we assessed policy support intentions (α = 0.91) and attitudes (α = 0.94). We measured bypassing beliefs, which was assessed by averaging two items from not at all to very much so. These items include, Genetically modified (GM) crops are beneficial for bee populations. The production of genetically modified (GM) crops is key to saving our bee populations. The reliability of the measure was good (α = 0.80).

#### Analysis

We utilized ANOVA with planned contrasts to assess experimental effects on all outcome variables.

### Experiment 3

#### Design and participants

In this preregistered study (https://aspredicted.org/blind.php?x=BJJ_3DSwe), we recruited 772 participants from Dynata (www.dynata.com) for a between-subjects experiment with four conditions. Each participant was asked to read two articles of relatively equal word length (217–220 words). Procedures were identical to Experiments 1 and 2, except that participants were randomized to one of four conditions: (1) bypassing condition, (2) correction condition, (3) misinformation control, and (4) true control. Participants in the first three conditions read an article containing misinformation about GM foods. In Experiment 3, the misinformation article discusses a (debunked) study on mice that claimed GM foods may lead to accelerated tumor growth. For those in the correction condition, participants then read an article that directly addressed the misinformation in the previous article. The article utilized best practices by providing an alternative explanation and factual refutation of the misinformation. Participants in the bypassing condition read an article identical to Experiment 2 describing the potential for GM crops to save the bee population. For those in the misinformation control condition, participants then read a control article identical to Experiment 2 on airport traveling. Lastly, those in the true control condition read two articles unrelated to GM foods focused on community parks and airport travel. After reading the two articles, all participants completed our dependent measures. Lastly, all participants were debriefed about the study and read a statement providing correct information about GM foods and directly refuted the misinformation they read in the first article.

Supplementary Table 1 indicates sample characteristics in comparison to estimates by the 2021 US Census for sex, race/ethnicity, and education. Compared to the census estimates, our sample had slightly more female participants and slightly fewer participants who identified as Hispanic or Latino and who attained less than a high school education. About 59% of participants identified as female, 41% identified as male, and less than 1% identified as non-binary or preferred not to answer. On average, participants were 44.67 (SD = 25.97) years old, most were married (48%), and the median income was $40,000 to $49,999. Further, the sample was politically moderate (M = 3.08, SD = 1.27); about 40% identified as a Republican while 30% identified as a Democrat. Although many participants indicated no religion or religious beliefs (32%), about 20% identified as Protestant and 23% identified as Roman Catholic.

#### Measures

The measurement procedures in this experiment were the same as in prior experiments except for the measurement of belief in misinformation and the bypassing beliefs, which were measured with a dichotomous choice of either *Agree* or *Disagree* to better visualize the impact of different strategies through alternative measure options. We examined belief in misinformation using a 1-item measure with the question, *The genetically modified (GM) corn causes the acceleration of tumor growth in rats*. Further, we also measured bypassing beliefs (α = 0.66) with items identical to Experiment 2 except with the dichotomous answer choice of *Agree* or *Disagree*.

#### Analysis

For policy support intentions and attitudes toward policies, we utilized ANOVA with planned contrasts to assess all outcome variables. Due to the dichotomous nature of the belief items, we utilized logistic regression with the misinformation control condition as the reference group to assess differences in belief in misinformation and bypassing beliefs.

## Supplementary Information

Supplementary Information.

## Data Availability

The datasets analyzed during the current study are available in the Open Science Framework repository, https://osf.io/rgw84/?view_only=18c30e212c97419e95e917e326e810a5.
