# Supplementary material for: Bypassing misinformation without confrontation improves policy support as much as correcting it
Source: Sci Rep. 2023 Apr 12;13:6005. doi: 10.1038/s41598-023-33299-5 (PMC10097652; doi:10.1038/s41598-023-33299-5)
Supplement: Supplementary file 1 — Supplementary Information. [file 41598_2023_33299_MOESM1_ESM.docx]

# Supplementary Information

## Supporting Information

**Belief in True Information.** We also measured beliefs in true information (see Table S2). For Experiments 1 and 2, beliefs in true information were assessed by averaging three items from not at all to very much so. These items include, *The reports of allergic reactions due to genetically modified (GM) corn have been shown to be false / The allergic reactions from genetically modified (GM) corn are due to the corn containing bacteria / The allergic reactions from genetically modified (GM) corn food products are not due to the genetic modification per se.* The reliability of the measures was satisfactory (Study 1, α =.80; Study 2, α =.82), and results were analyzed using ANOVA with planned contrasts. We find results consistent with our main findings that the correction condition significant improved true beliefs compared to the misinformation control.

For Experiment 3, we preregistered true beliefs (see Table S3), which were assessed by averaging three items with answer choices of *Agree* or *Disagree*. These items include, *The reports of accelerated tumor growth due to genetically modified (GM) corn have been shown to be false. /* *The accelerated tumor growth from rats feed with genetically modified (GM) corn were due to the specific breed of rats. /* *The finding that genetically modified (GM) corn accelerated tumor growth was found to be inconclusive.* The reliability of the measure was adequate (α =.71), and results were analyzed using logistic regression. Consistent with Experiment 1 and 2, the correction condition significantly improved true beliefs compared to the misinformation control.

**Mediation Analyses.**

The mechanism of bypassing was experimentally tested by redirecting misinformation recipients to alternate beliefs about the policy. Although we did not preregister mediation analyses, we conducted supplementary analyses in Experiments 2 and 3, both of which introduced new beliefs that were significantly affected by bypassing. The mediational analyses were conducted using path analysis with the Lavaan R package (Rosseel, 2012). The results showed a significant indirect effect (*ab* = -0.383, *p* < .001, 95% CI: [-0.574, -0.199]) in Experiment 2 and a nonsignificant but directionally consistent effect (*ab* = -0.048, *p* = .097, 95% CI: [-0.108, 0.007]) in Experiment 3. Future studies powered for mediational analyses should revisit this issue while also considering that the effects of bypassing may be more complex. For example, message recipients are well known to develop idiosyncratic responses to the content of persuasive communications (Greenwald, 1968; Petty et al., 1981), as a result of which other beliefs suggested by a bypassing message could mediate the impact of bypassing on attitudes and policy support. In addition, a bypassing message may affect the evaluations of the outcome it describes, by, for example, influencing evaluations of the benefits of supporting bee populations (see Albarracín, 2002; Fishbein & Ajzen, 1975). Future research on bypassing should investigate these possibilities.

**Table S1.** Sample Characteristics for Study 3 (N = 772).

Characteristic *n* % 2021 Census (%)

Female 437 58.6 50.5

Race/Ethnicity

Non-Hispanic White 472 63.3 59.3

Black 116 15.5 13.6

Hispanic or Latino 95 12.7 18.9

Asian 40 5.4 6.1

Other (AI/AN, NH/PI, 2+ races) 40 5.4 4.5

Education

HS or less 28 3.8 10.7

HS graduate/GED 214 28.7 26.3

Some college 163 21.8 19.3

Associate's/Bachelor's degree 259 34.7 30.0

Advanced degree 80 10.7 13.8

*Note.* About 26 participants did not report any demographic information, and 2 participants reported “Other” for education attained.

**Table S2.** Means and Standard Deviations for Belief in Accurate Information (Experiments 1 and 2).

|  | Misinformation & Bypassing  *M* (SD) | Misinformation & Correction  *M* (*SD*) | Misinformation Only (Control) *M* (SD) | F (dfs) | *p* |
| --- | --- | --- | --- | --- | --- |
| Experiment 1 (N = 360) | 2.46 ^a^  (0.85) | 3.72 ^b^  (1.12) | 2.48 ^a^  (0.94) | 65.24 (2, 357) | <.001 |
| Experiment 2 (N = 303) | 2.48 ^a^  (0.98) | 3.73 ^b^  (1.24) | 2.23 ^a^  (0.98) | 56.83 (2, 300) | <.001 |

*Note.* Different superscripts indicate statistically significant pairwise contrasts.

**Table S3.** Logistic Regression Results for Belief in Accurate Information (Experiment 3; N = 772).

|  | B | SE | *z* | *p* | OR [95% CI] |
| --- | --- | --- | --- | --- | --- |
| Misinformation & Bypassing | .24 | .21 | 1.12 | .262 | 1.27 [.84, 1.92] |
| Misinformation & Correction | 1.14 | .22 | 5.32 | <.001 | 3.14 [2.07, 4.80] |
| No Misinformation Control | .56 | .21 | 2.68 | .007 | 1.75 [1.16, 2.64] |
| Constant | -.54 | .15 | -3.56 | <.001 |  |

*Note.* The reference category is the misinformation control condition.

**References**

Albarracín, D. (2002). Cognition in persuasion: An analysis of information processing in response to persuasive communications. In *Advances in Experimental Social Psychology* (Vol. 34, pp. 61–130). Academic Press. https://doi.org/10.1016/S0065-2601(02)80004-1

Fishbein, M., & Ajzen, I. (1975). *Belief, Attitude, Intention, and Behavior: An Introduction to Theory and Research*. Addison-Wesley Publishing Company.

Greenwald, A. G. (1968). Cognitive learning, cognitive response to persuasion, and attitude change. In *Psychological foundations of attitudes* (pp. 147–170). Elsevier.

Petty, R. E., Ostrom, T. M., & Brock, T. C. (1981). *Cognitive Responses in Persuasion*. L. Erlbaum Associates.

Rosseel, Y. (2012). lavaan: An R package for structural equation modeling. *Journal of Statistical Software*, *48*(2), 1–36.
